# Bis{2-bromo-4-chloro-6-[(*E*)-(2,6-di­methyl­phen­yl)imino­meth­yl]phenolato-κ^2^ *N*,*O*}cobalt(II)

**DOI:** 10.1107/S1600536813003747

**Published:** 2013-02-20

**Authors:** Gang Zhu, Bao-Juan Jiao

**Affiliations:** a School of Chemistry and Chemical Engineering, Xi’an University of Arts and Science, Xi’an, Shaanxi 710065, People’s Republic of China

## Abstract

In the title complex, [Co(C_15_H_12_BrClNO)_2_], the Co^II^ ion is coordinated by two *N*,*O*-bidentate 2-bromo-4-chloro-6-[(*E*)-(2,6-dimethyl­phen­yl)imino­meth­yl]phenolate ligands, generating a squashed CoN_2_O_2_ tetra­hedral coordination geometry. The dihedral angles between the aromatic rings in the ligands are 82.60 (14) and 71.79 (14)°. The complex has approximate local noncrystallographic twofold symmetry. In the crystal, weak aromatic π–π stacking is observed [centroid–centroid separation = 3.6434 (18) Å].

## Related literature

For background to Schiff bases, see: Billson *et al.* (2000 ▶); Carlton *et al.* (1995 ▶); Feng *et al.* (2008 ▶); Liu *et al.* (2009 ▶).
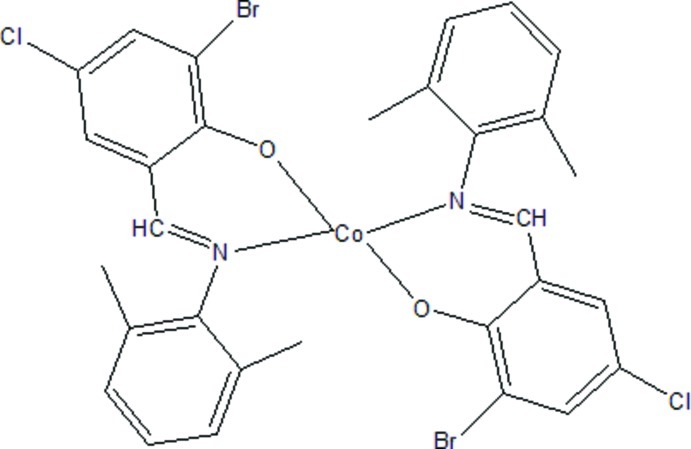


## Experimental

### 

#### Crystal data

[Co(C_15_H_12_BrClNO)_2_]
*M* *_r_* = 734.16
Monoclinic, 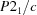

*a* = 11.608 (2) Å
*b* = 24.157 (4) Å
*c* = 11.354 (2) Å
β = 114.380 (2)°
*V* = 2899.8 (9) Å^3^
*Z* = 4
Mo *K*α radiation
μ = 3.56 mm^−1^
*T* = 153 K
0.49 × 0.27 × 0.12 mm

#### Data collection

Rigaku AFC10/Saturn724+ diffractometer
Absorption correction: multi-scan (*CrystalClear*; Rigaku, 2008 ▶) *T*_min_ = 0.274, *T*_max_ = 0.683
32947 measured reflections
7718 independent reflections
6813 reflections with *I* > 2σ(*I*)
*R*_int_ = 0.047

#### Refinement

*R*[*F* ^2^ > 2σ(*F* ^2^)] = 0.044
*wR*(*F* ^2^) = 0.119
*S* = 1.00
7718 reflections
356 parameters
H-atom parameters constrained
Δρ_max_ = 0.54 e Å^−3^
Δρ_min_ = −0.63 e Å^−3^

### 

Data collection: *CrystalClear* (Rigaku, 2008 ▶); cell refinement: *CrystalClear*; data reduction: *CrystalClear*; program(s) used to solve structure: *SHELXS97* (Sheldrick, 2008 ▶); program(s) used to refine structure: *SHELXL97* (Sheldrick, 2008 ▶); molecular graphics: *SHELXTL* (Sheldrick, 2008 ▶); software used to prepare material for publication: *SHELXL97*.

## Supplementary Material

Crystal structure: contains datablock(s) I, global. DOI: 10.1107/S1600536813003747/hb7032sup1.cif

Structure factors: contains datablock(s) I. DOI: 10.1107/S1600536813003747/hb7032Isup2.hkl

Additional supplementary materials:  crystallographic information; 3D view; checkCIF report

## Figures and Tables

**Table 1:** Selected bond lengths (Å)

Co1—O2	1.9006 (18)
Co1—O1	1.9144 (18)
Co1—N2	1.983 (2)
Co1—N1	2.000 (2)

**Fig. 1.**
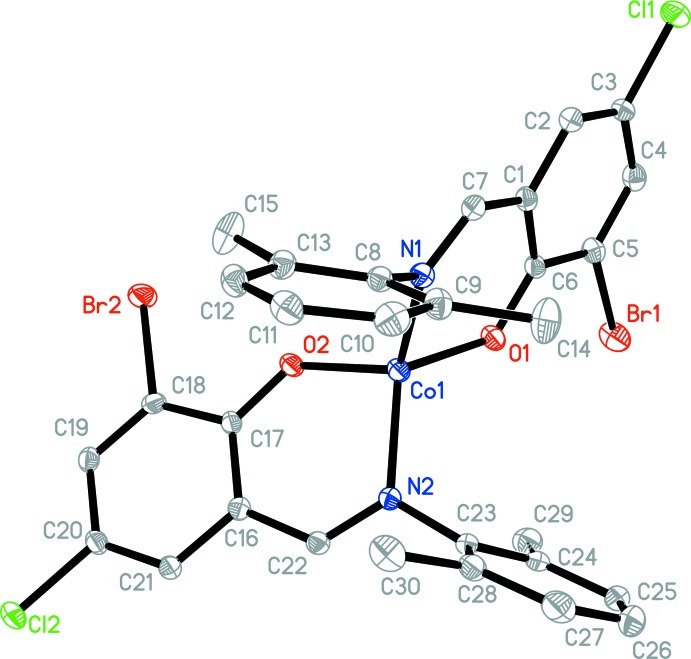
The structure of the title complex in 30% probability ellipsoids. H atoms are deleted for clarity.

**Fig. 2.**
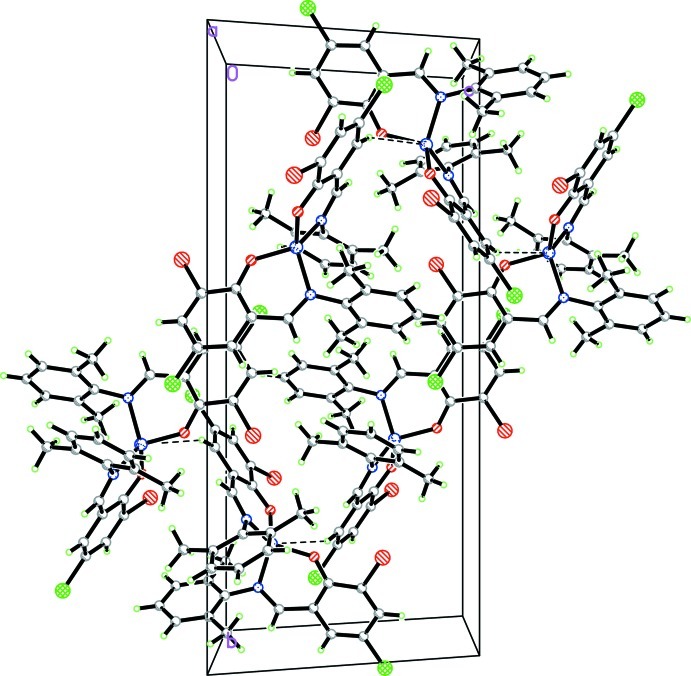
The crystal packing of the title complex, viewed along the a axis.

## supplementary crystallographic information

### Comment

Schiff bases possess strong coordination function and may act as bi-, tri-, and poly-dentate ligands to yield plenty of mono-, bi-, tri-, and poly-nuclear Schiff-base complexes, so the design of metal-organic coordination polymers is of current interest in the fields of supramolecular chemistry and crystal engineering because of their potential applications as functional materials (Feng, *et al.*, 2008; Liu, *et al.*, 2009). Meanwhile, Schiff bases and their metal complexes exhibit biological activity as antibiotics, antiviral and antitumour agents because of their specific structures (Billson, *et al.*, 2000; Carlton, *et al.*, 1995). Thus, it is quite important to have a good understanding of the structure of such metal complexes.

In this paper, we report synthesis and crystal structure of a new cobalt(II) complex, bis[2-((*E*)-(2,6-dimethylphenylimino) methyl)-6-bromo-4-chlorophenol]cobalt(II). The structure of the complex had been established accurately from the X-ray single-crystal diffraction study. The Co(II) ion in the monomeric unit seems to reside in a distorted tetrahedral environment and bonds to two oxygen atoms and two nitrogen atoms from two Schiff bases.

A thermal ellipsoid drawing and crystal packing structure of the title complex are shown in Figure 1 and Figuer 2. The Co(C_15_H_12_BrClNO)_2_ motif is asymmetrical with Co—N and Co—O bonds, the corresponding distances are *d*(Co—N1) = 2.000 (2), *d*(Co—O1) = 1.9144 (18), *d*(Co—N2) = 1.983 (2), *d*(Co—O2) = 1.9006 (2)Å. The cobalt atom is in a distorted tetrahedral environmente, where the values of *trans* bond angles also indicate the coordination environment with O(2)—Co(1)—N(1), O(2)—Co(1)—N(2) and O(2)—Co(1)—O(1) angles of 116.97 (9), 94.94 (8) and 113.77 (8) °, respectively.

In the crystal structure of the complex the atoms of the phenyl ring plane A (C(1) C(2) C(3) C(4) C(5) C(6)) and the chelate ring formed by the same ligand plane B ((O(1)/Co(1)/N(1)/C(7)/C(1)/C(6)) are nearly coplanar with a dihedral angle of 8.09 (10) °. Because of the conjugation effects through the imino double bond N(1)═C(7), the phenyl ring A and the phenyl ring C (C(8) C(9) C(10) C(11) C(12) C(13)) joined by the N(1)═C(7) bond, are non-coplanar (with the dihedral angle 74.55 (7) °, decreasing the steric effects between the two rings. If the two chelate planes, planes B and D((N(2)/Co(1)/O(2)/C(17)/C(16)/C(22)), are compared the dihedral angle is 87.19 (6) °, naerly perpendicular.

### Experimental

3-Bromo-5-chlorosalicylaldehyde (0.2 mmol, 47.2 mg) and 2,6-Dimethylaniline (0.2 mmol, 24.2 mg) were dissolved in EtOH (15 mL). The mixture was stirred for 30 min at room temperature to give an orange solution. To the resulting orange solution was added Co(CH_3_COO)_2_ (0.1 mmol, 17.7 mg). Then a clear brown solution was obtained. The title complex were obtained from the solution after 4 d in the form of red chunks. The product was filtered, washed with EtOH, and dried over anhydrous CaCl_2_*in vacuo* overnight. Yield: 70%. Anal. Calcd. (%) for C_30_H_24_CoN_2_O_2_Cl_2_Br_2_: C, 49.08; H, 3.29; N, 3.82. Found (%): C, 49.13; H, 3.43; N, 3.70.

### Crystal data

**Table:**

[Co(C_15_H_12_BrClNO)_2_]	*F*(000) = 1460
*M**_r_* = 734.16	*D*_x_ = 1.682 Mg m^−^^3^
Monoclinic, *P*2_1_/*c*	Mo *K*α radiation, λ = 0.71073 Å
*a* = 11.608 (2) Å	Cell parameters from 10097 reflections
*b* = 24.157 (4) Å	θ = 2.1–29.1°
*c* = 11.354 (2) Å	µ = 3.56 mm^−^^1^
β = 114.380 (2)°	*T* = 153 K
*V* = 2899.8 (9) Å^3^	Chunk, red
*Z* = 4	0.49 × 0.27 × 0.12 mm

### Data collection

**Table:**

Rigaku AFC10/Saturn724+ diffractometer	7718 independent reflections
Radiation source: Rotating Anode	6813 reflections with *I* > 2σ(*I*)
Graphite monochromator	*R*_int_ = 0.047
Detector resolution: 28.5714 pixels mm^-1^	θ_max_ = 29.1°, θ_min_ = 2.3°
phi and ω scans	*h* = −15→15
Absorption correction: multi-scan (*CrystalClear*; Rigaku, 2008)	*k* = −33→32
*T*_min_ = 0.274, *T*_max_ = 0.683	*l* = −15→15
32947 measured reflections	

### Refinement

**Table:**

Refinement on *F*^2^	Primary atom site location: structure-invariant direct methods
Least-squares matrix: full	Secondary atom site location: difference Fourier map
*R*[*F*^2^ > 2σ(*F*^2^)] = 0.044	Hydrogen site location: inferred from neighbouring sites
*wR*(*F*^2^) = 0.119	H-atom parameters constrained
*S* = 1.00	*w* = 1/[σ^2^(*F*_o_^2^) + (0.0651*P*)^2^ + 0.516*P*] where *P* = (*F*_o_^2^ + 2*F*_c_^2^)/3
7718 reflections	(Δ/σ)_max_ = 0.002
356 parameters	Δρ_max_ = 0.54 e Å^−^^3^
0 restraints	Δρ_min_ = −0.63 e Å^−^^3^

### Special details

**Table:**

Geometry. All e.s.d.'s (except the e.s.d. in the dihedral angle between two l.s. planes) are estimated using the full covariance matrix. The cell e.s.d.'s are taken into account individually in the estimation of e.s.d.'s in distances, angles and torsion angles; correlations between e.s.d.'s in cell parameters are only used when they are defined by crystal symmetry. An approximate (isotropic) treatment of cell e.s.d.'s is used for estimating e.s.d.'s involving l.s. planes.
Refinement. Refinement of *F*^2^ against ALL reflections. The weighted *R*-factor *wR* and goodness of fit *S* are based on *F*^2^, conventional *R*-factors *R* are based on *F*, with *F* set to zero for negative *F*^2^. The threshold expression of *F*^2^ > σ(*F*^2^) is used only for calculating *R*-factors(gt) *etc*. and is not relevant to the choice of reflections for refinement. *R*-factors based on *F*^2^ are statistically about twice as large as those based on *F*, and *R*- factors based on ALL data will be even larger.

### Fractional atomic coordinates and isotropic or equivalent isotropic displacement parameters (Å^2^)

**Table:**

	*x*	*y*	*z*	*U*_iso_*/*U*_eq_	
Co1	0.26654 (3)	0.662257 (15)	0.69368 (3)	0.02274 (10)	
Br1	0.57530 (3)	0.644847 (13)	1.13829 (3)	0.03965 (10)	
Br2	−0.09274 (3)	0.764024 (13)	0.69082 (3)	0.04195 (10)	
Cl1	0.36329 (7)	0.44165 (3)	1.15246 (7)	0.03762 (18)	
Cl2	−0.01848 (7)	0.91557 (3)	0.36421 (7)	0.03602 (17)	
O1	0.37754 (18)	0.64192 (7)	0.86650 (17)	0.0264 (4)	
O2	0.14215 (17)	0.71526 (7)	0.68464 (18)	0.0272 (4)	
N1	0.2067 (2)	0.58493 (9)	0.6390 (2)	0.0234 (5)	
N2	0.34280 (19)	0.70873 (9)	0.60062 (19)	0.0208 (4)	
C1	0.2975 (2)	0.54925 (11)	0.8618 (2)	0.0231 (5)	
C2	0.2949 (2)	0.50225 (11)	0.9335 (3)	0.0270 (6)	
H2	0.2416	0.4720	0.8913	0.032*	
C3	0.3687 (2)	0.49968 (11)	1.0639 (3)	0.0277 (6)	
C4	0.4516 (3)	0.54284 (12)	1.1261 (3)	0.0287 (6)	
H4	0.5049	0.5406	1.2158	0.034*	
C5	0.4548 (3)	0.58833 (11)	1.0562 (2)	0.0259 (6)	
C6	0.3753 (2)	0.59526 (10)	0.9230 (2)	0.0225 (5)	
C7	0.2230 (2)	0.54617 (11)	0.7227 (3)	0.0254 (5)	
H7	0.1819	0.5119	0.6900	0.030*	
C8	0.1440 (2)	0.57063 (11)	0.5034 (2)	0.0239 (5)	
C9	0.2103 (3)	0.53997 (11)	0.4467 (3)	0.0291 (6)	
C10	0.1533 (3)	0.53156 (12)	0.3131 (3)	0.0326 (6)	
H10	0.1964	0.5108	0.2726	0.039*	
C11	0.0347 (3)	0.55301 (12)	0.2391 (3)	0.0320 (6)	
H11	−0.0025	0.5475	0.1481	0.038*	
C12	−0.0294 (3)	0.58239 (11)	0.2975 (3)	0.0297 (6)	
H12	−0.1105	0.5971	0.2456	0.036*	
C13	0.0222 (3)	0.59107 (12)	0.4314 (3)	0.0280 (6)	
C14	0.3415 (3)	0.51866 (15)	0.5239 (3)	0.0443 (8)	
H14A	0.3946	0.5487	0.5766	0.053*	
H14B	0.3773	0.5047	0.4651	0.053*	
H14C	0.3382	0.4886	0.5804	0.053*	
C15	−0.0532 (3)	0.62008 (16)	0.4928 (3)	0.0455 (8)	
H15A	−0.0562	0.5971	0.5626	0.055*	
H15B	−0.1394	0.6265	0.4277	0.055*	
H15C	−0.0134	0.6556	0.5282	0.055*	
C16	0.1773 (2)	0.77951 (10)	0.5396 (2)	0.0210 (5)	
C17	0.1093 (2)	0.75899 (10)	0.6102 (2)	0.0208 (5)	
C18	0.0010 (2)	0.78977 (11)	0.5987 (3)	0.0247 (5)	
C19	−0.0383 (2)	0.83669 (11)	0.5250 (3)	0.0244 (5)	
H19	−0.1116	0.8560	0.5198	0.029*	
C20	0.0307 (2)	0.85564 (11)	0.4578 (3)	0.0250 (5)	
C21	0.1359 (2)	0.82777 (10)	0.4642 (2)	0.0231 (5)	
H21	0.1815	0.8412	0.4173	0.028*	
C22	0.2909 (2)	0.75423 (11)	0.5415 (2)	0.0221 (5)	
H22	0.3315	0.7727	0.4952	0.027*	
C23	0.4569 (2)	0.68953 (11)	0.5936 (2)	0.0234 (5)	
C24	0.5715 (2)	0.69448 (11)	0.7039 (3)	0.0269 (6)	
C25	0.6783 (3)	0.67082 (13)	0.6988 (3)	0.0376 (7)	
H25	0.7568	0.6735	0.7721	0.045*	
C26	0.6736 (3)	0.64344 (14)	0.5902 (4)	0.0452 (9)	
H26	0.7476	0.6267	0.5900	0.054*	
C27	0.5609 (3)	0.64060 (13)	0.4820 (3)	0.0405 (8)	
H27	0.5586	0.6225	0.4067	0.049*	
C28	0.4499 (3)	0.66366 (12)	0.4805 (3)	0.0288 (6)	
C29	0.5791 (3)	0.72459 (13)	0.8230 (3)	0.0357 (7)	
H29A	0.5418	0.7016	0.8693	0.043*	
H29B	0.5325	0.7596	0.7977	0.043*	
H29C	0.6678	0.7322	0.8794	0.043*	
C30	0.3284 (3)	0.66150 (13)	0.3601 (3)	0.0387 (7)	
H30A	0.2577	0.6540	0.3838	0.046*	
H30B	0.3336	0.6320	0.3032	0.046*	
H30C	0.3148	0.6971	0.3148	0.046*	

### Atomic displacement parameters (Å^2^)

**Table:**

	*U*^11^	*U*^22^	*U*^33^	*U*^12^	*U*^13^	*U*^23^
Co1	0.02424 (18)	0.02062 (18)	0.02371 (19)	0.00174 (13)	0.01026 (15)	0.00289 (14)
Br1	0.04535 (19)	0.03466 (18)	0.02708 (17)	−0.00775 (13)	0.00302 (14)	−0.00110 (12)
Br2	0.03983 (18)	0.03857 (19)	0.0633 (2)	0.01156 (13)	0.03721 (17)	0.01945 (15)
Cl1	0.0409 (4)	0.0365 (4)	0.0354 (4)	0.0004 (3)	0.0157 (3)	0.0140 (3)
Cl2	0.0365 (4)	0.0288 (4)	0.0433 (4)	0.0091 (3)	0.0170 (3)	0.0161 (3)
O1	0.0289 (10)	0.0226 (10)	0.0251 (10)	0.0012 (7)	0.0084 (8)	0.0037 (8)
O2	0.0294 (10)	0.0232 (10)	0.0340 (10)	0.0063 (7)	0.0182 (8)	0.0091 (8)
N1	0.0228 (11)	0.0236 (11)	0.0233 (11)	−0.0004 (8)	0.0090 (9)	0.0003 (9)
N2	0.0201 (10)	0.0213 (11)	0.0214 (10)	0.0011 (8)	0.0091 (8)	0.0013 (8)
C1	0.0225 (12)	0.0230 (13)	0.0239 (13)	0.0043 (9)	0.0098 (10)	0.0019 (10)
C2	0.0248 (13)	0.0244 (14)	0.0317 (14)	0.0003 (10)	0.0115 (11)	0.0022 (11)
C3	0.0270 (13)	0.0263 (14)	0.0327 (15)	0.0033 (11)	0.0154 (12)	0.0093 (12)
C4	0.0299 (14)	0.0336 (15)	0.0229 (13)	0.0062 (11)	0.0113 (11)	0.0032 (11)
C5	0.0278 (13)	0.0241 (13)	0.0241 (13)	0.0021 (10)	0.0089 (11)	−0.0011 (11)
C6	0.0227 (12)	0.0237 (13)	0.0232 (13)	0.0044 (9)	0.0115 (10)	0.0038 (10)
C7	0.0213 (12)	0.0255 (13)	0.0287 (14)	0.0000 (10)	0.0096 (11)	0.0010 (11)
C8	0.0238 (12)	0.0243 (13)	0.0229 (13)	−0.0013 (10)	0.0091 (11)	0.0014 (10)
C9	0.0307 (14)	0.0244 (14)	0.0301 (15)	0.0008 (11)	0.0105 (12)	−0.0026 (11)
C10	0.0367 (16)	0.0277 (15)	0.0345 (16)	−0.0033 (12)	0.0160 (13)	−0.0094 (12)
C11	0.0378 (16)	0.0294 (15)	0.0218 (13)	−0.0041 (12)	0.0052 (12)	−0.0018 (11)
C12	0.0280 (14)	0.0270 (14)	0.0255 (14)	−0.0005 (11)	0.0022 (11)	−0.0002 (11)
C13	0.0259 (13)	0.0290 (15)	0.0270 (14)	−0.0015 (10)	0.0088 (11)	−0.0016 (11)
C14	0.0354 (17)	0.050 (2)	0.0422 (18)	0.0135 (14)	0.0113 (15)	−0.0085 (16)
C15	0.0268 (15)	0.065 (2)	0.0403 (18)	0.0040 (15)	0.0095 (14)	−0.0139 (17)
C16	0.0206 (12)	0.0188 (12)	0.0225 (12)	−0.0001 (9)	0.0078 (10)	−0.0020 (10)
C17	0.0200 (11)	0.0193 (12)	0.0210 (12)	0.0011 (9)	0.0063 (10)	0.0008 (10)
C18	0.0221 (12)	0.0251 (13)	0.0300 (14)	−0.0015 (10)	0.0139 (11)	0.0014 (11)
C19	0.0199 (12)	0.0235 (13)	0.0275 (14)	0.0032 (9)	0.0076 (10)	0.0008 (11)
C20	0.0257 (13)	0.0215 (13)	0.0248 (13)	0.0018 (10)	0.0075 (11)	0.0038 (10)
C21	0.0225 (12)	0.0228 (13)	0.0227 (13)	−0.0001 (10)	0.0080 (10)	0.0019 (10)
C22	0.0226 (12)	0.0227 (13)	0.0210 (12)	−0.0004 (9)	0.0090 (10)	0.0013 (10)
C23	0.0222 (12)	0.0239 (13)	0.0271 (13)	0.0026 (10)	0.0133 (11)	0.0075 (11)
C24	0.0239 (13)	0.0269 (14)	0.0310 (14)	−0.0002 (10)	0.0123 (11)	0.0118 (11)
C25	0.0246 (14)	0.0439 (18)	0.0441 (18)	0.0050 (12)	0.0140 (13)	0.0217 (15)
C26	0.0390 (18)	0.048 (2)	0.061 (2)	0.0186 (14)	0.0329 (17)	0.0210 (17)
C27	0.057 (2)	0.0370 (18)	0.0435 (18)	0.0131 (15)	0.0372 (17)	0.0081 (14)
C28	0.0340 (15)	0.0265 (14)	0.0303 (15)	0.0056 (11)	0.0178 (12)	0.0062 (11)
C29	0.0310 (15)	0.0383 (17)	0.0314 (16)	−0.0085 (12)	0.0066 (13)	0.0018 (13)
C30	0.0503 (19)	0.0364 (17)	0.0291 (16)	0.0005 (14)	0.0162 (14)	−0.0037 (13)

### Geometric parameters (Å, º)

**Table:**

Co1—O2	1.9006 (18)	C13—C15	1.499 (4)
Co1—O1	1.9144 (18)	C14—H14A	0.9800
Co1—N2	1.983 (2)	C14—H14B	0.9800
Co1—N1	2.000 (2)	C14—H14C	0.9800
Br1—C5	1.901 (3)	C15—H15A	0.9800
Br2—C18	1.898 (3)	C15—H15B	0.9800
Cl1—C3	1.742 (3)	C15—H15C	0.9800
Cl2—C20	1.746 (3)	C16—C21	1.408 (3)
O1—C6	1.302 (3)	C16—C17	1.426 (4)
O2—C17	1.308 (3)	C16—C22	1.445 (3)
N1—C7	1.291 (3)	C17—C18	1.420 (3)
N1—C8	1.447 (3)	C18—C19	1.370 (4)
N2—C22	1.299 (3)	C19—C20	1.392 (4)
N2—C23	1.437 (3)	C19—H19	0.9500
C1—C2	1.405 (4)	C20—C21	1.370 (4)
C1—C6	1.420 (4)	C21—H21	0.9500
C1—C7	1.456 (4)	C22—H22	0.9500
C2—C3	1.372 (4)	C23—C28	1.400 (4)
C2—H2	0.9500	C23—C24	1.406 (4)
C3—C4	1.397 (4)	C24—C25	1.387 (4)
C4—C5	1.365 (4)	C24—C29	1.506 (4)
C4—H4	0.9500	C25—C26	1.381 (5)
C5—C6	1.420 (4)	C25—H25	0.9500
C7—H7	0.9500	C26—C27	1.378 (5)
C8—C13	1.399 (4)	C26—H26	0.9500
C8—C9	1.402 (4)	C27—C28	1.397 (4)
C9—C10	1.397 (4)	C27—H27	0.9500
C9—C14	1.500 (4)	C28—C30	1.506 (4)
C10—C11	1.383 (4)	C29—H29A	0.9800
C10—H10	0.9500	C29—H29B	0.9800
C11—C12	1.381 (4)	C29—H29C	0.9800
C11—H11	0.9500	C30—H30A	0.9800
C12—C13	1.400 (4)	C30—H30B	0.9800
C12—H12	0.9500	C30—H30C	0.9800
			
O2—Co1—O1	113.77 (8)	H14B—C14—H14C	109.5
O2—Co1—N2	94.94 (8)	C13—C15—H15A	109.5
O1—Co1—N2	115.58 (8)	C13—C15—H15B	109.5
O2—Co1—N1	116.97 (9)	H15A—C15—H15B	109.5
O1—Co1—N1	94.53 (8)	C13—C15—H15C	109.5
N2—Co1—N1	122.51 (9)	H15A—C15—H15C	109.5
C6—O1—Co1	125.79 (17)	H15B—C15—H15C	109.5
C17—O2—Co1	126.25 (17)	C21—C16—C17	120.0 (2)
C7—N1—C8	117.8 (2)	C21—C16—C22	116.1 (2)
C7—N1—Co1	121.47 (18)	C17—C16—C22	123.9 (2)
C8—N1—Co1	120.72 (17)	O2—C17—C18	119.5 (2)
C22—N2—C23	119.1 (2)	O2—C17—C16	124.3 (2)
C22—N2—Co1	122.70 (18)	C18—C17—C16	116.2 (2)
C23—N2—Co1	118.14 (16)	C19—C18—C17	123.2 (2)
C2—C1—C6	120.7 (2)	C19—C18—Br2	119.1 (2)
C2—C1—C7	116.2 (2)	C17—C18—Br2	117.72 (19)
C6—C1—C7	122.9 (2)	C18—C19—C20	119.0 (2)
C3—C2—C1	120.6 (3)	C18—C19—H19	120.5
C3—C2—H2	119.7	C20—C19—H19	120.5
C1—C2—H2	119.7	C21—C20—C19	120.7 (2)
C2—C3—C4	120.3 (2)	C21—C20—Cl2	120.0 (2)
C2—C3—Cl1	120.3 (2)	C19—C20—Cl2	119.2 (2)
C4—C3—Cl1	119.4 (2)	C20—C21—C16	120.8 (2)
C5—C4—C3	119.0 (2)	C20—C21—H21	119.6
C5—C4—H4	120.5	C16—C21—H21	119.6
C3—C4—H4	120.5	N2—C22—C16	126.2 (2)
C4—C5—C6	123.5 (3)	N2—C22—H22	116.9
C4—C5—Br1	119.0 (2)	C16—C22—H22	116.9
C6—C5—Br1	117.4 (2)	C28—C23—C24	122.0 (3)
O1—C6—C5	119.4 (2)	C28—C23—N2	119.3 (2)
O1—C6—C1	125.1 (2)	C24—C23—N2	118.5 (2)
C5—C6—C1	115.6 (2)	C25—C24—C23	117.4 (3)
N1—C7—C1	127.0 (2)	C25—C24—C29	121.0 (3)
N1—C7—H7	116.5	C23—C24—C29	121.6 (3)
C1—C7—H7	116.5	C26—C25—C24	121.9 (3)
C13—C8—C9	122.2 (2)	C26—C25—H25	119.1
C13—C8—N1	118.7 (2)	C24—C25—H25	119.1
C9—C8—N1	119.0 (2)	C27—C26—C25	119.5 (3)
C10—C9—C8	118.1 (2)	C27—C26—H26	120.2
C10—C9—C14	119.8 (3)	C25—C26—H26	120.2
C8—C9—C14	122.0 (2)	C26—C27—C28	121.5 (3)
C11—C10—C9	120.8 (3)	C26—C27—H27	119.2
C11—C10—H10	119.6	C28—C27—H27	119.2
C9—C10—H10	119.6	C27—C28—C23	117.6 (3)
C12—C11—C10	119.9 (3)	C27—C28—C30	120.7 (3)
C12—C11—H11	120.0	C23—C28—C30	121.7 (3)
C10—C11—H11	120.0	C24—C29—H29A	109.5
C11—C12—C13	121.7 (3)	C24—C29—H29B	109.5
C11—C12—H12	119.2	H29A—C29—H29B	109.5
C13—C12—H12	119.2	C24—C29—H29C	109.5
C8—C13—C12	117.2 (3)	H29A—C29—H29C	109.5
C8—C13—C15	122.5 (2)	H29B—C29—H29C	109.5
C12—C13—C15	120.3 (2)	C28—C30—H30A	109.5
C9—C14—H14A	109.5	C28—C30—H30B	109.5
C9—C14—H14B	109.5	H30A—C30—H30B	109.5
H14A—C14—H14B	109.5	C28—C30—H30C	109.5
C9—C14—H14C	109.5	H30A—C30—H30C	109.5
H14A—C14—H14C	109.5	H30B—C30—H30C	109.5
			
O2—Co1—O1—C6	106.0 (2)	C14—C9—C10—C11	−177.0 (3)
N2—Co1—O1—C6	−145.6 (2)	C9—C10—C11—C12	−1.0 (5)
N1—Co1—O1—C6	−16.2 (2)	C10—C11—C12—C13	−0.3 (4)
O1—Co1—O2—C17	135.3 (2)	C9—C8—C13—C12	−3.5 (4)
N2—Co1—O2—C17	14.5 (2)	N1—C8—C13—C12	172.1 (2)
N1—Co1—O2—C17	−115.9 (2)	C9—C8—C13—C15	175.2 (3)
O2—Co1—N1—C7	−102.8 (2)	N1—C8—C13—C15	−9.2 (4)
O1—Co1—N1—C7	16.8 (2)	C11—C12—C13—C8	2.5 (4)
N2—Co1—N1—C7	141.1 (2)	C11—C12—C13—C15	−176.1 (3)
O2—Co1—N1—C8	77.5 (2)	Co1—O2—C17—C18	169.79 (18)
O1—Co1—N1—C8	−162.86 (19)	Co1—O2—C17—C16	−11.6 (4)
N2—Co1—N1—C8	−38.5 (2)	C21—C16—C17—O2	−178.5 (2)
O2—Co1—N2—C22	−10.4 (2)	C22—C16—C17—O2	0.2 (4)
O1—Co1—N2—C22	−129.8 (2)	C21—C16—C17—C18	0.1 (4)
N1—Co1—N2—C22	116.1 (2)	C22—C16—C17—C18	178.8 (2)
O2—Co1—N2—C23	172.64 (18)	O2—C17—C18—C19	178.6 (2)
O1—Co1—N2—C23	53.3 (2)	C16—C17—C18—C19	−0.1 (4)
N1—Co1—N2—C23	−60.8 (2)	O2—C17—C18—Br2	−1.4 (3)
C6—C1—C2—C3	−0.8 (4)	C16—C17—C18—Br2	179.94 (18)
C7—C1—C2—C3	175.6 (2)	C17—C18—C19—C20	−0.2 (4)
C1—C2—C3—C4	−2.7 (4)	Br2—C18—C19—C20	179.80 (19)
C1—C2—C3—Cl1	179.1 (2)	C18—C19—C20—C21	0.4 (4)
C2—C3—C4—C5	2.2 (4)	C18—C19—C20—Cl2	−179.7 (2)
Cl1—C3—C4—C5	−179.6 (2)	C19—C20—C21—C16	−0.4 (4)
C3—C4—C5—C6	1.9 (4)	Cl2—C20—C21—C16	179.75 (19)
C3—C4—C5—Br1	−175.9 (2)	C17—C16—C21—C20	0.1 (4)
Co1—O1—C6—C5	−173.34 (18)	C22—C16—C21—C20	−178.7 (2)
Co1—O1—C6—C1	6.3 (4)	C23—N2—C22—C16	−179.5 (2)
C4—C5—C6—O1	174.4 (3)	Co1—N2—C22—C16	3.6 (4)
Br1—C5—C6—O1	−7.7 (3)	C21—C16—C22—N2	−177.4 (2)
C4—C5—C6—C1	−5.2 (4)	C17—C16—C22—N2	3.8 (4)
Br1—C5—C6—C1	172.68 (19)	C22—N2—C23—C28	−76.9 (3)
C2—C1—C6—O1	−175.1 (3)	Co1—N2—C23—C28	100.1 (2)
C7—C1—C6—O1	8.8 (4)	C22—N2—C23—C24	106.8 (3)
C2—C1—C6—C5	4.5 (4)	Co1—N2—C23—C24	−76.1 (3)
C7—C1—C6—C5	−171.6 (2)	C28—C23—C24—C25	−2.3 (4)
C8—N1—C7—C1	171.2 (2)	N2—C23—C24—C25	173.9 (2)
Co1—N1—C7—C1	−8.4 (4)	C28—C23—C24—C29	177.2 (3)
C2—C1—C7—N1	176.6 (3)	N2—C23—C24—C29	−6.6 (4)
C6—C1—C7—N1	−7.2 (4)	C23—C24—C25—C26	0.1 (4)
C7—N1—C8—C13	110.2 (3)	C29—C24—C25—C26	−179.4 (3)
Co1—N1—C8—C13	−70.2 (3)	C24—C25—C26—C27	1.8 (5)
C7—N1—C8—C9	−74.1 (3)	C25—C26—C27—C28	−1.6 (5)
Co1—N1—C8—C9	105.6 (3)	C26—C27—C28—C23	−0.5 (5)
C13—C8—C9—C10	2.2 (4)	C26—C27—C28—C30	178.3 (3)
N1—C8—C9—C10	−173.4 (2)	C24—C23—C28—C27	2.5 (4)
C13—C8—C9—C14	179.3 (3)	N2—C23—C28—C27	−173.7 (2)
N1—C8—C9—C14	3.7 (4)	C24—C23—C28—C30	−176.3 (3)
C8—C9—C10—C11	0.1 (4)	N2—C23—C28—C30	7.6 (4)
